# Cisd2 Preserves the Youthful Pattern of the Liver Proteome during Natural Aging of Mice

**DOI:** 10.3390/biomedicines9091229

**Published:** 2021-09-15

**Authors:** Chen-Hua Huang, Yi-Long Huang, Zhao-Qing Shen, Chao-Hsiung Lin, Ting-Fen Tsai

**Affiliations:** 1 Department of Life Sciences and Institute of Genome Sciences, National Yang Ming Chiao Tung University, Taipei 112, Taiwan; denny1210.y@nycu.edu.tw (C.-H.H.); yilonghuang@nycu.edu.tw (Y.-L.H.); d40008003@ym.edu.tw (Z.-Q.S.); 2 Aging and Health Research Center, National Yang Ming Chiao Tung University, Taipei 112, Taiwan; 3 Institute of Molecular and Genomic Medicine, National Health Research Institutes, Zhunan 350, Taiwan

**Keywords:** Cisd2, non-alcoholic fatty liver disease, aging, lipid metabolism, mitochondria, protein homeostasis

## Abstract

Cisd2 (CDGSH iron sulfur domain 2) is a pro-longevity gene that extends the lifespan and health span of mice, ameliorates age-associated structural damage and limits functional decline in multiple tissues. Non-alcoholic fatty liver disease (NAFLD), which plays an important role in age-related liver disorders, is the most common liver disease worldwide. However, no medicines that can be used to specifically and effectively treat NAFLD are currently approved for this disease. Our aim was to provide pathological and molecular evidence to show that Cisd2 protects the liver from age-related dysregulation of lipid metabolism and protein homeostasis. This study makes four major discoveries. Firstly, a persistently high level of Cisd2 protects the liver from age-related fat accumulation. Secondly, proteomics analysis revealed that Cisd2 ameliorates age-related dysregulation of lipid metabolism, including lipid biosynthesis and β-oxidation, in mitochondria and peroxisomes. Thirdly, Cisd2 attenuates aging-associated oxidative modifications of proteins. Finally, Cisd2 regulates intracellular protein homeostasis by maintaining the functionality of molecular chaperones and protein synthesis machinery. Our proteomics findings highlight Cisd2 as a novel molecular target for the development of therapies targeting fatty liver diseases, and these new therapies are likely to help prevent subsequent malignant progression to cirrhosis and hepatocellular carcinoma.

## 1. Introduction

Aging is a process during which organisms gradually lose their ability to carry out normal functions and maintain homeostasis. The extent of age-associated perturbations in the structure and function of each organ is site-specific, indicating that aging is a multidimensional process in organisms [1]. The liver is a vital metabolic organ that orchestrates the energy metabolism of the whole body. Specifically in the liver, age-associated fat accumulation and dysregulated lipid metabolism compromise normal hepatic function and lead to fatty liver, which is accompanied by histopathological damage [2].

There is accumulating evidence pointing toward an increased prevalence of nonalcoholic fatty liver disease (NAFLD) among older humans [3]. NAFLD is characterized by excessive lipid accumulation in hepatocytes. Although it is not very dangerous at an early stage, a significant portion of affected individuals develop a severe form of liver disease known as nonalcoholic steatohepatitis (NASH) which can progress to cirrhosis and hepatocellular carcinoma (HCC). The complex pathophysiology underlying the development and progression of NASH remains incompletely understood. However, many studies have indicated that hepatic mitochondria play a crucial role in NAFLD; functional decline in these mitochondria promotes metabolic disturbances that appear to contribute to NAFLD progression [4].

Mitochondrial function is crucial in the physiopathology of NAFLD. In mammals, the main substrates that an organism uses to generate ATP and maintain metabolic homeostasis are glucose and fatty acids. In this context, fatty acids may be used for highly efficient energy storage and overall they deliver more energy per gram than carbohydrates like glucose. The catabolic process involving fatty acids occurs primarily in the mitochondrial matrix, where fatty acids are broken down to generate acetyl-CoA by β-oxidation. In the liver, increased mitochondrial biogenesis [5], TCA cycle [6] and β-oxidation [7] through increase of free fatty acid (FFA) availability [8] have been shown to be involved in the pathogenesis of NAFLD at an early stage. Notably, these changes in mitochondrial metabolism are thought to reflect an adaption to the increase in intrahepatic lipid handling induced by NAFLD [9].

Previous studies have suggested that reactive oxygen species (ROS) and reactive nitrogen species (RNS) also play a pivotal role in the progression of NAFLD [10]. ROS are generated as byproducts of the electron transport chain (ETC) in the mitochondria. Notably, an elevated level of ROS is usually generated under pathological conditions when the ETC is impaired. Remarkably, previous studies have revealed that the efficiency of mitochondrial respiration among NASH patients is significantly decreased, possibly due to the leakage of electrons out of the ETC and the uncoupling of oxidation and phosphorylation [5]. Therefore, under chronic FFA overload, mitochondria generate huge amounts of ROS, which further damage mitochondria, and this forms a vicious cycle. When the antioxidant defense mechanism fails to win the fight against the augmented oxidative stress, the fatty liver progresses to NASH, which is dangerous and is associated with significant liver damage and inflammation [5].

Cisd2 (CDGSH iron sulfur domain 2) is a pro-longevity gene which plays a crucial role in lifespan control [11]. Our previous studies have demonstrated that a persistently high level of Cisd2 leads to a 19.4% and 18.8% increase in the median lifespans of male and female mice, respectively [12]. In humans, the functioning of neurological, musculoskeletal and cardiovascular systems decline as an individual ages [1]. Intriguingly, in Cisd2 transgenic (Cisd2TG) mice, an increased level of Cisd2 delays aging and ameliorates age-related functional decline in multiple tissues and organs, including the brain, skeletal muscles, neurons, skin and heart [12,13,14]. Conversely, in the Cisd2 knockout (Cisd2KO) mice, Cisd2 deficiency causes a premature phenotype and shortens their lifespan [15]. Intriguingly, Cisd2 haploinsufficiency promotes fat accumulation in hepatocytes and predisposes mice to the development of NAFLD [16]. Regarding subcellular location, Cisd2 is primarily located in the mitochondrial outer membranes, the ER and the mitochondria-associated ER membranes [17]. In addition, our previous study has revealed that Cisd2 interacts with Serca2 to modulate intracellular Ca^2+^ homeostasis; Serca2 is a calcium pump that can catalyze the hydrolysis of ATP to transport Ca^2+^ from the cytosol into the ER [17]. Furthermore, Cisd2 haploinsufficiency leads to a significant increase in basal cytosolic Ca^2+^ and an overload in mitochondrial Ca^2+^ content, thereby compromising mitochondrial respiration via a reduction in the oxygen consumption rate of the liver [16]. By way of contrast, a persistently high level of Cisd2 in the Cisd2TG mice preserves mitochondrial function and protects the activity of Complexes I–V in the skeletal muscles of old mice [12]. Moreover, using the Western pattern diet-induced NAFLD, the levels of reactive species, including ROS and RNS, are significantly increased in the livers of heterozygous mice carrying hepatocyte-specific knockout of Cisd2 (Cisd2hKO+/–) while overexpression of Cisd2 alleviates the Western pattern diet-induced oxidative stress in Cisd2TG mice [18].

The protective function of Cisd2 in the liver prompted us to investigate whether an enhanced level of Cisd2 can improve the age-related dysregulation associated with lipid metabolism and fatty liver. Here, we applied a proteomic study to pinpoint the molecular mechanisms that are involved in lipid biosynthesis and catabolism in terms of their biological processes and cellular compartments. Furthermore, we carried out a detailed proteomics analysis of differentially expressed proteins (DEPs) in the naturally aged wild-type (WT) mice and compared the results with long-lived Cisd2TG mice carrying four copies of the Cisd2 gene; these long-lived Cisd2TG mice have a twofold overexpression of Cisd2 in their livers. So far, very few proteomics studies have focused on the beneficial effects of longevity genes on liver aging. Thus, this study may lead to the discovery of the critical regulators and/or pathways that play important roles in liver aging and should help the development of therapeutic strategies aimed at treating fatty liver disease, thereby preventing a subsequent malignant progression to cirrhosis and HCC.

## 2. Materials and Methods

### 2.1. Mice

The Cisd2TG mice carrying four copies of the Cisd2 gene and expressing a twofold increase in the Cisd2 protein were generated as previously described [16]. Male C57BL/6 mice were used for all the experiments in this study. The mice were maintained at 21 ± 1 °C with a 12 h light/dark cycle and had free access to food and water under specific pathogen-free conditions. To study the effect of Cisd2 on the age-related dysregulation of lipid metabolism in the liver, the experiments were performed using the following mice: wild-type (WT) mice at a young age (3-month-old; 3-mo) and an old age (26-mo), as well as Cisd2TG mice at an old age (26-mo). The body weights and liver weights of the mice were measured after the mice had been euthanized. All the animal protocols were approved by the Institutional Animal Care and Use Committee (IACUC) of the National Yang Ming Chiao Tung University (No. 1080410, 19 April 2019).

### 2.2. Liver Histopathology

Liver tissue samples were collected and fixed in a 10% buffered formalin solution before undergoing tissue processing and paraffin embedding. Hematoxylin and eosin (H&E) staining was carried out on liver sections (3–4 μm) and this was conducted using standard protocols. Lipid accumulation was analyzed by Oil Red O staining (O0625, Sigma-Aldrich, Munich, Germany) using optimal cutting temperature-embedded cryosections.

### 2.3. Immunohistochemistry

Immunohistochemistry (IHC) staining was carried out on paraffin-embedded liver sections (3 μm) using an anti-glutamine synthetase antibody (610518, BD Bioscience, San Jose, CA, USA), which was followed by counterstaining with hematoxylin. In brief, the sections were deparaffinized, rehydrated and antigen-retrieved using a target retrieval solution (S1699, Dako, Carpinteria, CA, USA). Subsequently, the liver sections were quenched using 3% H_2_O_2_ in PBS, blocked using 5% bovine serum albumin in PBS and incubated with a primary antibody in an antibody diluent (ab64211, Abcam, Cambridge, UK). The target protein in the liver sections was finally detected using an LSAB kit (K0679, Dako, Carpinteria, CA, USA) and visualized using the Chemicon IHC Select System (DAB150, Millipore, Burlington, MA, USA) according to the manufacturer’s instructions.

### 2.4. Western Blotting

Liver tissues were homogenized in a lysis buffer (100 mM NaCl, 50 mM Tris at pH 7.4, 1 mM EDTA, 1% Triton X-100 with protease inhibitor and phosphatase inhibitor cocktails, Roche) and denatured in a 2% SDS sample buffer (50 mM Tris at pH 6.8, 2% SDS, 100 mM dithiothreitol and 10% glycerol) for 10 min at 100 °C. The extracted proteins were separated by SDS–polyacrylamide gel electrophoresis and electrotransferred to a polyvinylidene fluoride membrane. The membranes were blocked with 5% (w/v) nonfat dried milk, probed with an antibody and detected with a Visualizer Kit (WBKLS0500, Millipore, Burlington, MA, USA). The following antibodies were used for Western blotting: S-sulfonated cysteine (ADI-OSA-820; Enzo Life Sciences, Ann Arbor, MI, USA), 3-nitrotyrosine (ab61392, Abcam, Cambridge, UK) and Gapdh (MAB374, Millipore, Burlington, MA, USA).

### 2.5. Liver Protein Extraction and Proteomics Analysis

Frozen liver samples were homogenized using a RIPA buffer containing protease inhibitors. The homogenate was centrifuged twice at 13,000× *g* for 20 min to remove insoluble cell debris. The final supernatant was collected and denatured in a sample buffer (2% SDS, 5% β-mercaptoethanol, 10% glycerol and 50 mM Tris HCl, pH 6.8) at 95 °C for 10 min before resolution with SDS–PAGE. In-gel digestion of gel pieces was performed with sequencing-grade trypsin (V5111, Promega, Mannheim, Germany) at 37 °C for 20 h. The tryptic peptides were extracted from gel by sonication in 50% ACN in a 0.1% trifluoroacetic acid aqueous solution. The extracted peptides were desalted by ZipTip (ZTC18M096, Millipore, Burlington, MA, USA) and analyzed with an Orbitrap Elite hybrid mass spectrometer (Thermo Electron, Bremen, Germany) equipped with a nanoAcquity system (Waters, Milford, MA, USA). The resulting samples were separated at a flow rate of 0.3 μL/min with a 210 min gradient from 5% to 35% acetonitrile with 0.1% formic acid. The mass spectra were acquired in the data-dependent acquisition mode. All the raw MS data were processed using the Proteome Discoverer (PD) software (version 2.4) (Thermo Fisher Scientific, Waltham, MA, USA) for protein identification and label-free quantification. The MS data were searched against the mouse Swiss-Prot database (www.uniprot.org, accessed on 12 August 2020) with a precursor mass tolerance set to 20 ppm and a fragment ion mass tolerance set to 0.8 Da. The identified proteins were included for further analysis when presenting with all of the three following parameters: (a) FDR for identified protein < 5%; (b) detected in at least > 50% samples; (c) at least one unique peptide was identified. A total of 3749 proteins were retained after the filtering.

### 2.6. Statistical Analysis

Comparisons between the three groups (3-mo WT, 26-mo WT, 26-mo Cisd2TG) were carried out using the one-way ANOVA *F*-test with the python package “scipy” and using post-hoc Tukey’s honestly significant difference (HSD) test with package “statsmodels”. The Benjamini and Hochberg procedure was performed to control the overall false discovery rate (FDR) of the multiple one-way ANOVA *F*-test. Tukey’s HSD test was conducted for the comparison between 26-mo WT vs. 3-mo WT as well as between 26-mo Cisd2TG vs. 26-mo WT after the differentially expressed proteins (DEPs) had been identified by the *F*-test. The significance level was defined as *p* < 0.05 when using Tukey’s HSD test. The proteomics dataset was loaded into the EZinfo 3.0.3 software (Umetrics, Umeå, Sweden) to allow principal component analysis (PCA). The levels of proteins were transformed into z-scores to allow presentation as heatmaps, which were generated by the Multi Experiment Viewer 4.9 software [19]. The 517 aging-associated DEPs, as well as the 319 DEPs that had expression changes reversed by Cisd2TG, were further analyzed for functional annotation using Gene Ontology (GO) via the PANTHER online tools (www.pantherdb.org, accessed on 23 May 2021). The significance level for each GO term was set at a fold enrichment > 2 and an FDR of < 5% for the GO biological process and cellular compartment analysis. The common enriched GO terms between 26-mo WT vs. 3-mo WT and 26-mo Cisd2TG vs. 26-mo WT were identified and then manually grouped into their annotated cellular functions.

## 3. Results

### 3.1. Cisd2 Attenuates Age-Related Fat Accumulation and Improves Histopathology in the Liver

**Figure 1:**
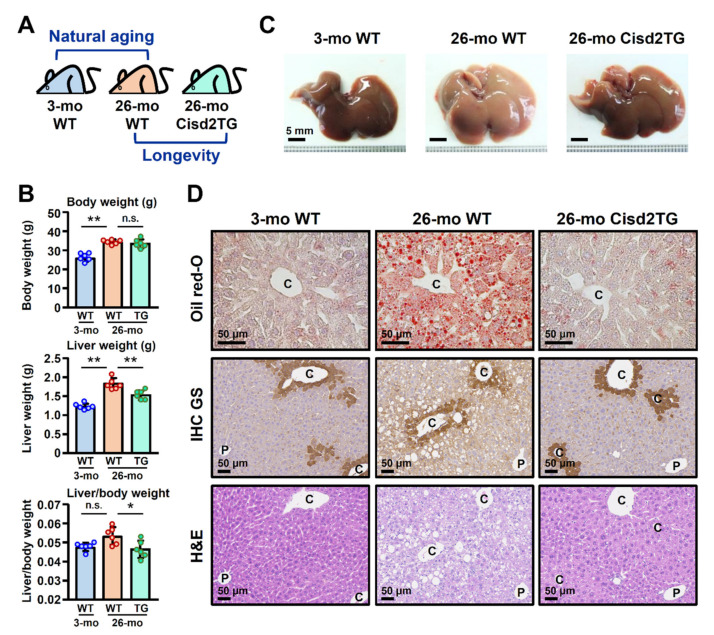
Cisd2 improves age-related fat accumulation and ameliorates the pathological abnormalities associated with natural aging. (**A**) Schematic representation of the study design used for liver proteome profiling. (**B**) Body weight, liver weight and the ratio of liver/body weight of the young WT mice (3-mo WT), old WT mice (26-mo WT) and old Cisd2TG mice (26-mo Cisd2TG) (*n* = 6 in each group). (**C**) Representative images of gross views of livers from the three groups. (**D**) Representative images of Oil Red O staining, IHC staining of glutamine synthetase (GS) and H&E staining of liver sections prepared from samples from the three mice groups. Hepatic steatosis around the central veins can be seen in the livers of the 26-mo WT mice. The data are presented as the means ± SD; * *p* < 0.05; ** *p* < 0.01 (Tukey’s HSD test after the one-way ANOVA had reached statistical significance). P, portal vein; C, central vein; n.s., non-significant.

Our previous study revealed that a half dose of the Cisd2 protein in mice is insufficient to carry out its normal functions in the liver, which can be described as a state of Cisd2 haploinsufficiency; these mice develop a phenotype similar to the clinical manifestations of NAFLD [16]. Intriguingly, it has been shown that enhanced expression of Cisd2 can decrease the levels of liver triglyceride and lipid peroxidation in the liver of HBx transgenic mice and attenuate the development of HBV-associated HCC [16]. Since dysregulation of lipid metabolism is a common phenotype of livers during natural aging, accordingly, we investigated whether enhanced Cisd2 expression can improve liver lipid metabolism and ameliorate age-related NAFLD. This study used two groups of mice and compared their liver tissues using pathological and proteomic analyses; these consisted of (1) a natural aging group that compared young WT mice (3-mo WT) with old WT mice (26-mo WT) and (2) a longevity group that compared old WT mice (26-mo WT) with old Cisd2TG mice (26-mo Cisd2TG) (Figure 1A).

When the 26-mo WT mice were examined, there was a significant increase in the weight of their livers, as well as an increase in the liver weight (Figure 1B). In addition, the morphology of the liver obtained from the 26-mo WT mice could be seen to be lighter in color and overall the livers looked larger. By way of contrast, when the Cisd2TG mice were examined, both weight and morphology of the livers were similar to those of the young mice (Figure 1C). In terms of histopathology, hepatic steatosis could be detected in the 26-mo WT mice as evidenced by Oil Red O staining of their liver sections. Intriguingly, in the Cisd2TG mice, a significant decrease in fat accumulation was found, while at the same time, the histology, as revealed by H&E staining of liver sections, was very similar to the results for the young mice (Figure 1D). Moreover, previous studies have shown that increased lipogenesis usually occurs around the central vein of the liver when NAFLD is present [16,20]. To examine whether Cisd2 overexpression attenuates abnormal lipid accumulation in the hepatocytes located around the central vein, we performed IHC staining of glutamine synthetase (GS), which is a marker used to identify pericentral hepatocytes. The results showed that, in the old WT mice (26-mo), most of the lipid droplets did indeed accumulate in the pericentral hepatocytes of their livers; however, we found a remarkable reduction in this lipid droplet deposition in the old Cisd2TG mice (Figure 1D). Taken together, these findings in the old mice indicate that Cisd2 appears to be able to protect the liver from age-related dysregulation of lipid metabolism and at the same time Cisd2 attenuates fat accumulation

### 3.2. The Youthful Pattern of the Liver Proteome Is Preserved in Old Cisd2 Transgenic Mice

**Figure 2:**
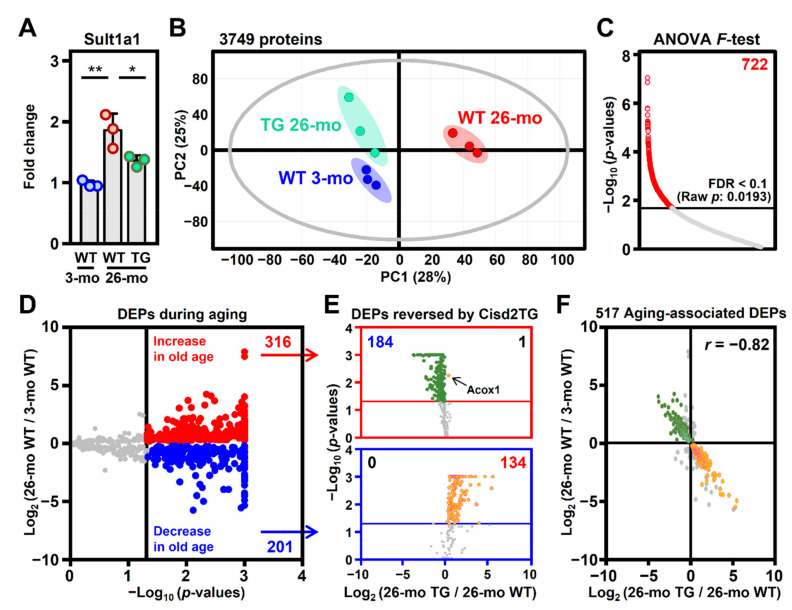
A youthful pattern of the liver proteome was maintained in the Cisd2 transgenic mice. (**A**) Levels of the Sult1a1 protein as a positive control for liver aging in the 3-mo WT, 26-mo WT and 26-mo Cisd2TG mice (*n* = 3 in each group); * *p* < 0.05; ** *p* < 0.01 (Tukey’s HSD test after the one-way ANOVA had reached statistical significance). (**B**) Principal component analysis (PCA) of the liver proteomes of the mice forming the three groups. (**C**) Differentially expressed proteins (DEPs) identified using the one-way ANOVA *F*-test, with the overall false discovery rate (FDR) controlled to be less than 10%. (**D**) Volcano plots showing selected aging-associated DEPs based on their *p*-values from Tukey’s HSD test after conducting the one-way ANOVA *F*-test. The vertical line denotes the significance threshold cutoff (*p*-value < 0.05). The proteins that passed this criterion are shown in either red (increased) or blue (decreased). (**E**) The DEPs reversed by Cisd2TG are identified among the 517 aging-associated DEPs based on the *p*-value of Tukey’s HSD test. The upper red box contains the 316 upregulated DEPs associated with aging, whereas the lower blue box contains the 201 downregulated DEPs associated with aging. The horizontal lines denote the significance threshold cutoff (*p*-value < 0.05). The proteins that had their changes in expression level reversed in the Cisd2TG mice are shown in either orange (increased in the Cisd2TG mice) or green (decreased in the Cisd2TG mice). (**F**) Pearson correlation analysis of the 517 aging-associated DEPs. The colored dots are the 319 aging-associated DEPs reversed in the Cisd2TG mice (184 aging up TG down; 134 aging down TG up).

In order to obtain insights into the mechanism(s) by which Cisd2 alleviates the age-related dysregulation of lipid metabolism, a label-free proteomics approach using LC–MS/MS was applied to investigate the molecular details and functional changes associated with the process. The protein level of Sult1a1 was used as a positive control for liver aging [21]. In the 26-mo WT mice, the level of Sult1a1 was found to be significantly increased; remarkably, in the 26-mo Cisd2TG mice, the level of Sult1a1 was significantly lower compared with that in the age and sex-matched WT mice (Figure 2A). Principal component analysis (PCA) of the global proteomic profiles revealed that there is a considerable separation between the profiles of the 26-mo WT mice and the 3-mo WT mice. Importantly, the group formed by the 26-mo Cisd2TG mice moved closer to the group of 3-mo WT mice, which indicates that the protein molecular profiles of the old Cisd2TG mice were quite different from those in the old WT mice and had significant similarity to those of the young mice (Figure 2B).

The differentially expressed proteins (DEPs) obtained from the two groups were first assessed using one-way ANOVA, and the overall false discovery rate (FDR) was controlled to be below 0.1 (Figure 2C). To further study the impact of the enhanced expression of the Cisd2 protein on the age-related alterations affecting the liver proteome, we analyzed the DEPs from naturally aged WT mice and long-lived Cisd2TG mice using post-hoc tests. A total of 517 proteins were identified as DEPs during natural aging (26-mo WT vs. 3-mo WT) (Figure 2D). Interestingly, among the 517 DEPs, the expression patterns of 319 DEPs were found to be reversed in terms of expression among the Cisd2TG mice. Specifically, the DEPs identified by comparing the 26-mo Cisd2TG and 26-mo WT mice all show an opposite trend to that which occurs during natural aging, and this trend is towards the pattern observed in the young mice (Figure 2E). Furthermore, a Pearson correlation analysis of the 517 DEPs also reveals a strong negative correlation (*r* = −0.82) between the fold changes related to longevity (26-mo Cisd2TG vs. 26-mo WT) and the fold changes related to natural aging (26-mo WT vs. 3-mo WT) (Figure 2F), which suggests that a persistently high level of the Cisd2 protein appears to slow down liver aging and helps to preserve a youthful proteome pattern in the livers of Cisd2TG mice.

**Figure 3:**
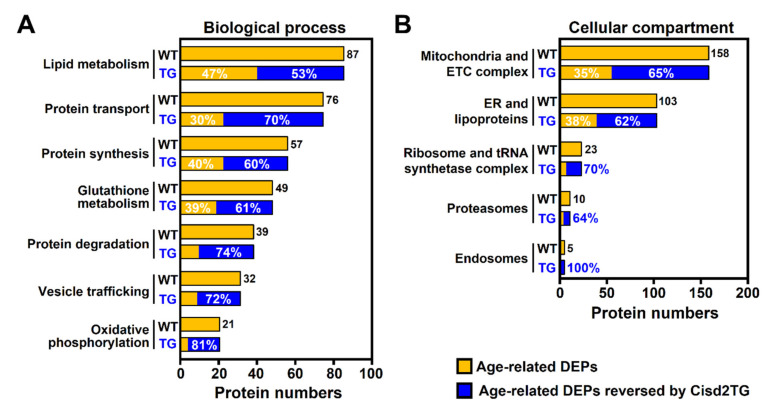
Age-related alterations in various biological processes and cellular compartments were attenuated in the livers of the Cisd2TG mice. Overrepresentation analysis (ORA) of the 517 DEPs during natural aging and the 319 DEPs reversed by Cisd2TG; this was carried out using PANTHER GO. (**A**) Biological process analysis. (**B**) Cellular compartment analysis. The common enriched GO terms between 26-mo WT vs. 3-mo WT mice and 26-mo Cisd2TG vs. 26-mo WT mice were identified and manually grouped into five or seven annotated cellular functions. The horizontal axis shows the numbers of aging-associated DEPs in each annotated cellular function. The aging-associated DEPs are labeled in orange, and the DEPs reversed by Cisd2TG are labeled in blue. The percentage in blue represents the coverage of the Cisd2-associated proteins in the aging-associated DEPs.

To investigate the age-related alterations in the liver in terms of biological processes and cellular compartments (organelles), the 517 DEPs associated with natural aging (26-mo WT vs. 3-mo WT) and the 319 DEPs which capable of being reverted by Cisd2TG were analyzed using the Gene Ontology (GO). Regarding the biological process analysis, the annotated cellular functions mainly involved lipid metabolism, protein homeostasis, glutathione metabolism, vesicle trafficking and oxidative phosphorylation. Notably, for each biological process, there was a significant percentage of proteins whose expression patterns were reversed in the Cisd2TG mice (Figure 3A). In the cellular compartment analysis, the main organelles identified in this analysis were mitochondria, the ER and lipoproteins, ribosomes and the tRNA synthetase complex, proteasomes and endosomes. Interestingly, the functions of these cellular organelles and complexes are consistent with the results of the biological process analysis. Importantly, for each cellular compartment or complex, more than 50% of the proteins involved were reversed in the Cisd2TG mice (Figure 3B).

### 3.3. Cisd2 Ameliorates Age-Related Dysregulation of Lipid Metabolism

Lipid metabolism in the liver involves lipid storage, lipid biosynthesis and β-oxidation. The formation of lipid droplets and metabolism of lipoproteins mainly take place in the ER; while lipid biosynthesis and transport largely take place in the cytoplasm. In addition, β-oxidation of fatty acids, which is the process by which fatty acids are broken down to produce energy, occurs in both mitochondria and peroxisomes. Mitochondria catalyze the β-oxidation of the majority of fatty acids, including short-chain (with aliphatic tails of 2–5 carbons), medium-chain (with aliphatic tails of 6–12 carbons), and long-chain (with aliphatic tails of 13–21 carbons) fatty acids. On the other hand, peroxisomes catalyze the β-oxidation of very-long-chain fatty acids with aliphatic tails of 22 or more carbons. Quantification of the proteins involved in all of these pathways was performed using the liver proteomic analysis.

**Figure 4:**
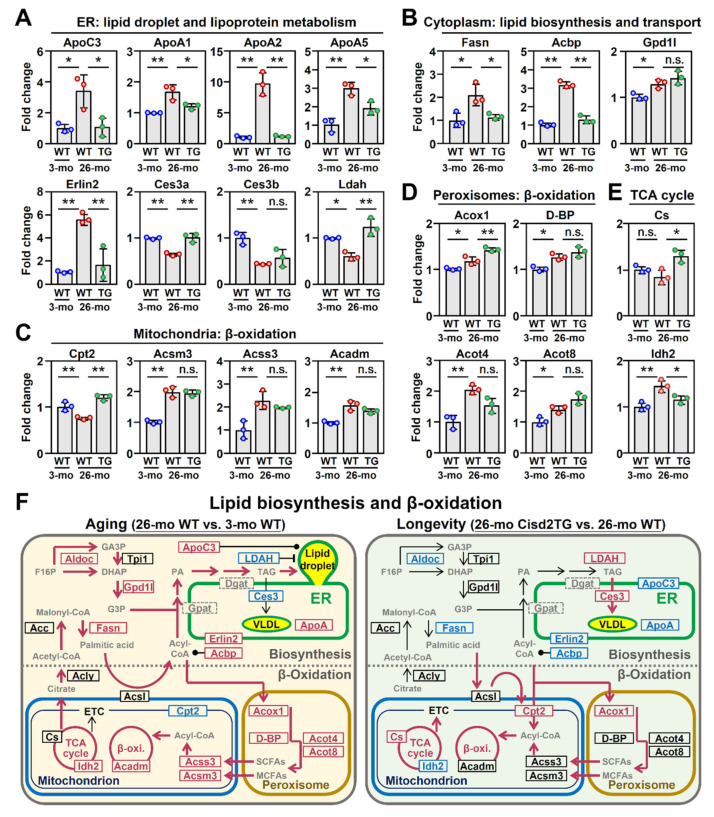
Cisd2 ameliorates age-related dysregulation of lipid metabolism in the liver. The aging-associated DEPs involved in the biological processes of (**A**) lipid droplet and lipoprotein metabolism, (**B**) lipid biosynthesis and transport, (**C**,**D**) mitochondrial and peroxisomal β-oxidation and (**E**) TCA cycle. Quantification of each protein is based on the relative intensity of the LC–MS results from three different mice from each group. The data are represented as the means ± SD; * *p* < 0.05; ** *p* < 0.01 (Tukey’s HSD test after the one-way ANOVA had reached statistical significance). (**F**) A schematic illustration of lipid metabolism in naturally aged WT mice and long-lived Cisd2TG mice. The proteins that are not identified in our datasets are marked by the dashed gray box. Red indicates upregulation and blue indicates downregulation of the proteins. A solid black dot represents a direct interaction between the protein and the metabolite (or lipid droplet). The bold arrows with red color indicate the predicted metabolic flux. n.s., non-significant.

In terms of the proteins involved in the formation of lipid droplets and the metabolism of lipoproteins, in the 26-mo WT mice, there was an increase in the level of apolipoprotein C3 (ApoC3), a lipid droplet-associated lipoprotein [22], as well as increased levels of three ApoA proteins (ApoA1, ApoA2, ApoA5). Remarkably, this dysregulated pattern of lipoproteins was reversed in the 26-mo Cisd2TG mice (Figure 4A). Interestingly, there was also a significant increase during natural aging of the WT mice in the level of ER lipid raft associated 2 (Erlin2), which plays an important role in lipogenesis [23]. Furthermore, this upregulated pattern of Erlin2 expression was reversed in the Cisd2TG mice. On the other hand, the levels of carboxylesterase 3/triacylglycerol hydrolase (Ces3) [24] and lipid droplet-associated hydrolase (Ldah) [25], which are lipid hydrolases that regulate the formation of lipid droplets, were significantly decreased during the natural aging of WT mice. Importantly, this downregulated pattern of Ces3 and Ldah expression was also found to be reversed in the old Cisd2TG mice (Figure 4A).

In terms of lipid biosynthesis, a significant increase in the level of two critical enzymes, namely of fatty acid synthase (Fasn) and Gpd1l, was found during the natural aging of the WT mice; however, only the change in the Fasn expression was reversed in the 26-mo Cisd2TG mice (Figure 4B). Furthermore, the level of the acyl-CoA binding protein (Acbp), which is involved in the maintenance of the intracellular pool of acyl-CoA as well as the transport of acyl-CoA, was found to be significantly increased in the 26-mo WT mice; furthermore, a similar and low level of Acbp was observed in both the 3-mo young mice and the 26-mo Cisd2TG mice (Figure 4B).

In terms of β-oxidation in the mitochondria, three of the proteins involved in the process, namely Acsm3, Acss3 and Acadm, were significantly increased in the old mice; however, only one protein, carnitine palmitoyltransferase 2 (Cpt2), which shuttles long-chain fatty acids into mitochondria [26], was found to be significantly decreased during the natural aging of the WT mice. Interestingly, Cpt2 was found to be significantly increased in the 26-mo Cisd2TG mice compared to the 26-mo WT mice (Figure 4C). In terms of β-oxidation in the peroxisome, four of the proteins involved in the pathway, namely Acox1, D-BP, Acot4 and Acot8, were found to be significantly increased during natural aging in both the WT and Cisd2TG mice (Figure 4D). The role of the peroxisome seems to be to act as an alternative pathway for the consumption of the huge backlog of acyl-CoA that can accumulate in the cytosol. The protective effect of the peroxisome during natural aging might explain why its key gene (acyl-CoA oxidase 1, Acox1), which is part of the peroxisomal β-oxidation [27], can be identified as an aging-associated DEP that stubbornly remains upregulated in the 26-mo Cisd2TG mice (shown in Figure 2E). Consequently, it seems likely that the upregulation of Cpt2 and Acox1 (Figure 4C,D) promotes the influx of acyl-CoA into mitochondria and peroxisomes, and this increase in β-oxidation might attenuate abnormal fat accumulation.

Taken together, the quantification results outlined above and obtained from an analysis of the liver proteome demonstrate that a high level of Cisd2 ameliorates the age-related dysregulation of lipid metabolism and that these molecular alterations in lipid metabolism are consistent with the pathological findings (Figure 1). A graphical summary comparing the lipid metabolism of livers from the naturally aged WT mice with those of the long-lived Cisd2TG mice is presented in Figure 4F.

### 3.4. Cisd2 Modulates the Redox Status, Calcium Homeostasis and Mitochondrial Respiration

**Figure 5:**
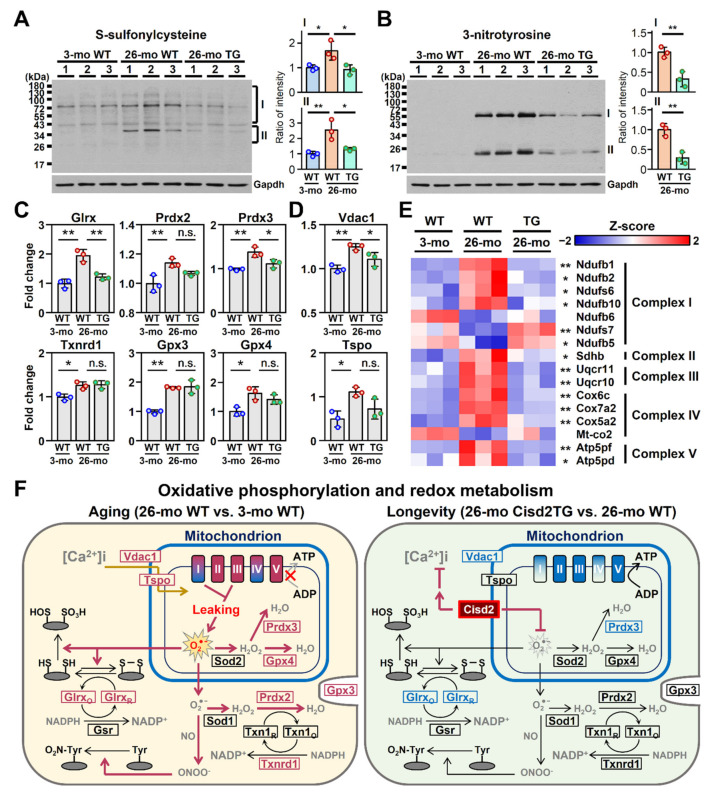
Cisd2 modulates redox status, calcium homeostasis and mitochondrial respiration. (**A**,**B**) Western blot analyses of the oxidative modification of liver proteins by S-sulfonylcysteine and 3-nitrotyrosine in the 3-mo WT, 26-mo WT and 26-mo Cisd2TG mice. Quantification of each sample is based on the entire intensities of each lane or region normalized against GAPDH; * *p* < 0.05; ** *p* < 0.01 (Tukey’s HSD test after the quantification of S-sulfonylcysteine and Student’s *t*-test after the quantification of 3-nitrotyrosine). (**C**,**D**) The aging-associated DEPs involved in calcium transport and the antioxidant response. The data are represented as the means ± SD; * *p* < 0.05; ** *p* < 0.01 (Tukey’s HSD test after the one-way ANOVA had reached statistical significance). (**E**) A heatmap revealing the difference between the 26-mo Cisd2TG and 26-mo WT mice in the aging-associated DEPs related to complexes I, II, III, IV and V of the mitochondrial ETC; * *p* < 0.05; ** *p* < 0.01 (Tukey’s HSD test). (**F**) A schematic illustration of redox status, calcium homeostasis and mitochondrial oxidative phosphorylation of the naturally aged WT mice and the long-lived Cisd2TG mice. Red indicates upregulation and blue indicates downregulation of the relevant proteins. n.s., non-significant. The “x” denotes the inhibited ATP synthesis during aging compared to the 26-mo Cisd2TG mice. The gray arrow was also used to emphasize this decreasing trend.

Our previous study revealed that Cisd2 plays an important role in maintaining a normal redox status [18]. To further analyze intracellular redox status and oxidative stress and evaluate the protective function of Cisd2 in the antioxidant defense during natural aging, we carried out Western blot analyses of liver extracts using antibodies targeting either cysteine S-sulfonation or tyrosine nitration. Notably, in the 26-mo WT mice, the overall levels of these two oxidative modifications of cellular proteins were found to be significantly increased. Remarkably, these oxidative modifications were found to be significantly attenuated in the 26-mo Cisd2TG mice (Figure 5A,B). Moreover, in the 26-mo WT mice, the levels of several antioxidant enzymes, namely Glrx, Prdx2, Prdx3, Txnrd1, Gpx3 and Gpx4, were also significantly increased; this was likely a compensatory effect in response to the elevated oxidative stress. Among these antioxidant enzymes, two of them, namely Glrx and Prdx3, were found to be downregulated in the 26-mo Cisd2TG mice compared to the 26-mo WT mice (Figure 5C).

In addition, one of our previous studies revealed that Cisd2 regulates calcium homeostasis and the mitochondrial function [11]. In this study, we carried out quantification of the proteins involved in these biological processes in the liver. In terms of calcium homeostasis, in the 26-mo WT mice, we found that there was a significant increase in the protein level of voltage-dependent anion-selective channel 1 (Vdac1), which is a major calcium ion transport channel and is located in the mitochondrial outer membrane. The upregulation of Vdac1 was also likely a compensatory effect in response to the elevated levels of cytosolic calcium caused by leakage of calcium from the ER during aging [28]. By way of contrast, in the 26-mo Cisd2TG mice, the upregulation of Vdac1 was significantly attenuated (Figure 5D).

Regarding the mitochondrial electron transfer chain (ETC), a total of 18 proteins were identified as DEPs in the liver during the natural aging of the WT mice. Strikingly, the changes in the expression patterns of the majority of these DEPs (14 proteins, 77%) were reversed in the 26-mo Cisd2TG mice; these changes moved the expression pattern towards a youthful one that is similar to that observed in the 3-mo young mice (Figure 5E). In addition, many of the dysregulated ETC proteins are the component proteins of complex I, which is where the majority of ROS production in the mitochondria takes place; this suggests that the increase in oxidative stress is probably attributable to malfunctioning of complexes I–V during oxidative phosphorylation, in particular of complex I. A pictorial summary comparing the redox status, calcium homeostasis and mitochondrial respiration of the livers of the naturally aged WT mice and the long-lived Cisd2TG mice is presented in Figure 5F.

### 3.5. Cisd2 Maintains Intracellular Protein Homeostasis

**Figure 6:**
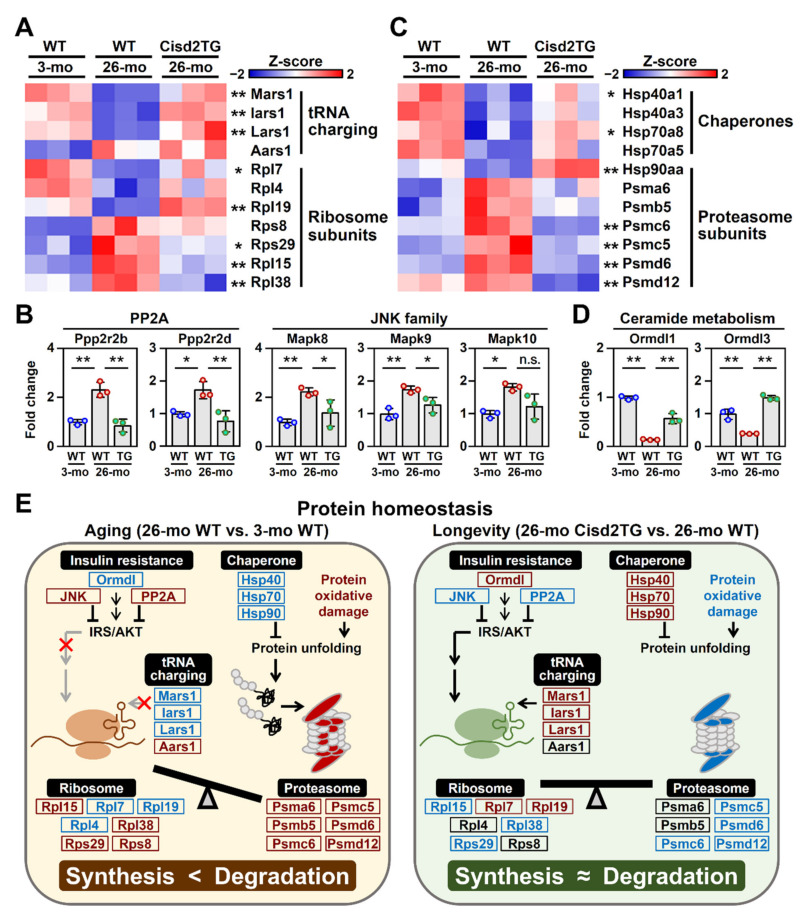
Cisd2 maintains protein homeostasis during aging. (**A**) A heatmap revealing the difference between the 26-mo Cisd2TG and 26-mo WT mice in the aging-associated DEPs related to protein synthesis; * *p* < 0.05; ** *p* < 0.01 (Tukey’s HSD test). (**B**) Upregulation of PP2A signaling and JNK signaling, both of which are involved in insulin resistance via inhibition of the IRS/AKT signaling. The data are presented as the means ± SD; * *p* < 0.05; ** *p* < 0.01 (Tukey’s HSD test after the one-way ANOVA had reached statistical significance). (**C**) A heatmap revealing the difference between the 26-mo Cisd2TG and 26-mo WT mice in the aging-associated DEPs related to protein degradation; * *p* < 0.05; ** *p* < 0.01 (Tukey’s HSD test). (**D**) The aging-associated DEPs involved in ceramide metabolism. The data are presented as the means ± SD; * *p* < 0.05; ** *p* < 0.01 (Tukey’s HSD test after the one-way ANOVA had reached statistical significance). (**E**) A schematic illustration of protein homeostasis in the naturally aged WT mice and the long-lived Cisd2TG mice. Red indicates upregulation and blue indicates downregulation of the relevant proteins. n.s., non-significant. The “x” denotes the inhibited IRS/AKT signaling and tRNA charging during aging compared to the 26-mo Cisd2TG mice. The gray arrows were also used to emphasize these decreasing trends.

The results of the GO analysis of the biological processes and cellular compartments revealed that protein homeostasis, including protein synthesis and transport, as well as ribosomal proteins and the tRNA synthetase complex, are all impaired during aging. Accordingly, we analyze the proteins involved in the biological process of translation, including the ribosome complex and tRNA ligases. Remarkably, in the 26-mo WT mice, quantification of the protein levels showed that there is a dysregulated pattern that affects many of the ribosomal subunits and tRNA ligases, that is, some of the proteins were abnormally increased, while the others were significantly decreased during aging (Figure 6A). We also analyzed the upstream signaling pathways of protein synthesis, namely PP2A and JNK signaling. Previous studies indicated that dysregulation of these two pathways results in insulin resistance via inhibition of the IRS/AKT signaling [29]. Notably, in the 26-mo WT mice, both PP2A and JNK signaling are significantly upregulated; however, in the Cisd2TG mice, the PP2A and JNK signaling was found to be less activated than in the 26-mo WT mice (Figure 6B).

We also investigated the DEPs involved in the process of protein degradation; these consisted of two groups of DEPs: (1) molecular chaperones that facilitate protein folding and are particularly required for the proper folding of large proteins and protein complexes and (2) components of proteasomes that are required for the degradation of unneeded or damaged proteins by proteolysis. Interestingly, in the 26-mo WT mice, a significant number of the molecular chaperones, namely Hsp40a1, Hsp70a8 and Hsp90aa, were significantly decreased during aging (Figure 6C); the consequence of these changes was likely to be an accumulation of misfolded proteins. Conversely, several subunits of the proteasome, namely Psmc6, Psmc5, Psmd6 and Psmd12, were found to be significantly increased (Figure 6C); these proteasome subunits were likely to be induced in response to the accumulation of misfolded proteins in the aged liver cells. Intriguingly, most of the dysregulations related to protein degradation seemed to be absent in the Cisd2TG mice (Figure 6C).

Additionally, previous studies indicated that sphingolipid ceramide inhibits insulin-stimulated glucose uptake and is one of the main factors involved in the generation of insulin resistance [30]. Strikingly, in the Cisd2TG mice, a higher level of Cisd2 appears to upregulate the expression of ORMDL sphingolipid biosynthesis regulators 1 and 3 (Ormdl1, Ormdl3) (Figure 6D), which inhibit the synthesis of ceramide thereby increasing insulin sensitivity in the liver [31]. A pictorial summary comparing the protein homeostasis between the livers of the naturally aged WT mice and the livers of the long-lived Cisd2TG mice is presented in Figure 6E.

## 4. Discussion

This study provides evidence in terms of liver histopathology and an analysis of liver proteomics that substantiate the hypothesis that Cisd2 protects the liver from age-related dysregulation of lipid metabolism. Several important discoveries are pinpointed in this study. Firstly, a persistently high level of Cisd2 protects the liver from age-related fat accumulation and reduces histopathological abnormalities in Cisd2TG mice. Secondly, the youthful pattern of the liver proteome is preserved in the Cisd2TG mice at an old age. In particular, this includes Cisd2 amelioration of the age-related dysregulation of lipid metabolism, including lipid biosynthesis and β-oxidation in the mitochondria and peroxisomes. As a result, abnormal fat accumulation is significantly improved via an inhibition of lipid biosynthesis and an acceleration of β-oxidation that consumes fatty acids generating energy. Thirdly, Cisd2 attenuates aging-associated oxidative modifications of proteins, modulates redox and calcium homeostasis and promotes mitochondrial respiration. Intriguingly, old Cisd2TG mice have levels of multiple component proteins of complexes I–V that remain well-preserved and seem to be maintained with a profile similar to that observed in young mice. Finally, Cisd2 maintains intracellular protein homeostasis. During the natural aging of WT mice, the protein synthesis machinery, namely ribosomal proteins and the tRNA synthetase complex, as well as various molecular chaperones, are impaired; this is accompanied by upregulation of the proteasomes involved in protein degradation. Importantly, most of these dysregulations affecting intracellular protein homeostasis are not detectable in the livers of Cisd2TG mice at an old age.

### 4.1. De Novo Lipogenesis and Lipid Droplet Homeostasis

Age has been correlated with a predisposition towards steatosis and NAFLD and this is accompanied by an increase in lipogenesis and lipotoxicity. Indeed, lipid metabolism was one of the most significantly altered metabolic pathways in the livers of the 26-mo WT mice (Figure 3A). As seen in Figure 4A,B, Fasn and Erlin2 significantly increased with age, and this effect was mitigated by enhanced expression of Cisd2. Erlin2, which is induced by a high-fat diet and fatty acid supplementation, had been previously shown to activate Srebp1c, which in turn upregulates Fasn and Dgat, and this promotes lipid biosynthesis in human liver cells [23]. Cisd2 overexpression seems to suppress aging-induced lipogenesis through inhibition of Erlin2 protein expression, at least in part. In addition, Cisd2 is able to suppress the age-dependent reduction in lipid droplet-associated hydrolase Ces3 and Ldah (Figure 4A,F), which is likely to also contribute to the improved lipid metabolism present in Cisd2TG mice. Elimination of hepatic Ces3 leads to lipid accumulation in the liver and decreased plasma VLDL levels [24]. Reduced VLDL secretion was also observed in the livers of heterozygous Cisd2 knockout mice during our previous study [16]. Thus, the suppression of de novo lipogenesis as well as an increase in hepatic lipid secretion might be the potential mechanisms underlying the Cisd2-mediated reduction in fat accumulation that occurs with aging.

### 4.2. The Compensatory Role of β-Oxidation in Aging-Induced NAFLD

Several changes in β-oxidation enzymes in response to aging were noted (Figure 4C,D). Age negatively regulates mitochondrial Cpt2, whereas peroxisomal Acox1 increases with age. Given that Cpt2 and Acox1 are rate-limiting, in terms of the enzymes involved in long-chain fatty acid β-oxidation, one would expect mitochondrial long-chain fatty acid β-oxidation to be compromised and peroxisomal β-oxidation to be activated during aging. Interestingly, increased Cisd2 is able to suppress the age-related reduction in Cpt2, while at the same time the age-related induction of Acox1 remains upregulated and, to an even higher extent, compared with that of old WT mice. Increasing evidence suggests that peroxisomes might exert a protective role and help to compensate for excessive hepatic lipid accumulation during the development of fatty liver [32]. For example, Acox1-deficient mice and mice with hepatocyte-selective elimination of peroxisomes both exhibit spontaneous steatosis, steatohepatitis and the development of HCC [27,33]. Thus, aging may result in a metabolic shift from mitochondrial to peroxisomal β-oxidation, while Cisd2 enables efficient fatty acid oxidation through both of these two metabolism pathways.

Although β-oxidation of long-chain fatty acids is compromised due to the decreased level of Cpt2 during aging, we found that proteins involved in fatty acid activation and mitochondrial β-oxidation (Acsm3, Acss3 and Acadm) were significantly increased in the old WT mice (Figure 4C). These results are consistent with previous findings; these showed that an increase in mitochondrial β-oxidation was observed at the early stages of NAFLD [7] and that it is characterized by excessive fat accumulation in hepatocytes without overt inflammation affecting the liver (Figure 1D). Interestingly, the substrates of these upregulated enzymes during aging are members of the short-chain and medium-chain fatty acids, which are mainly produced by β-oxidation in peroxisomes. This suggests that peroxisomes and mitochondria may coordinate their functions and act together to prevent lipid accumulation and that the increase in β-oxidation is likely to be a compensatory effect and/or an adaptive mechanism in response to fat accumulation [34].

### 4.3. Cisd2 Regulates Oxidative Stress and the Antioxidant Response

Hepatic β-oxidation is coupled to the TCA cycle and mitochondrial respiration and is responsible for the generation of ROS, which can be detrimental to the survival of cells under normal physiological conditions. Specifically, overactive β-oxidation will overload the hepatic mitochondrial ETC with reducing equivalents and this in turn will accelerate ROS production. In NAFLD, chronic FFA overload within the liver increases β-oxidation, thus promoting oxidative stress and inducing antioxidant responses [8,9].

The Cisd2 protein contains a redox-active 2Fe–2S cluster in its CDGSH domain, which suggests that it may be involved in the regulation of cellular ROS levels. In the study, we found that Cisd2 ameliorates age-associated oxidative damage to various liver proteins (Figure 5A,B). In particular, the age-dependent increases of Glrx and Prdx3 were significantly normalized in livers of the long-lived Cisd2TG mice (Figure 5C). Glrx-deficient mice spontaneously develop hepatic steatosis [35], whereas Prdx3 overexpression in mitochondria is able to effectively decrease cellular ROS [36]. However, Cisd2-mediated redox regulation during old age cannot be solely explained by the reduction in Glrx and Prdx3 levels. It would therefore be of great interest to identify how Cisd2 helps to preserve antioxidant capacity without increasing the expression of the various proteins that participate in redox homeostasis.

### 4.4. Cisd2 Preserves Mitochondrial ETC and Ribosomal Protein Complexes

Mitochondrial dysfunction has been implicated in various aspects of aging. However, the effect of age on the expression of mitochondrial ETC subunits is less clear. Most of the DEPs associated with Complexes II–V were upregulated. In contrast, some of the subunits belonging to Complex I were upregulated, but some were downregulated at an advanced age (Figure 5E). Therefore, impaired mitochondria may be attributable to the disruption of the stoichiometric balance among components, although the assembly of mitochondrial complexes cannot be easily revealed by our current proteomic approach. Likewise, such a mosaic pattern of protein regulation cans also be detected in the ribosome subunits during aging (Figure 6A). Importantly, such age-dependent alterations of the OXPHOS complexes and translation machinery were generally less pronounced in the long-lived Cisd2TG mice. We propose that Cisd2 preserves coordination of the ETC and ribosomal protein complexes. Nevertheless, quantitative analyses for the mitochondrial functions and enzymatic activities of ETC complexes are necessary to get new insights into the role of Cisd2 in the mitochondria during natural aging of the liver, which require fresh tissues obtained from 24–26-month-old WT and Cisd2TG mice in the future.

### 4.5. IRS/AKT Signaling during Liver Aging

Loss of protein homeostasis (proteostasis) is one of the central hallmarks of aging. Aging seems to disrupt various proteostasis modules, including ribosomal proteins, tRNA synthetase complexes, chaperones and proteasomes. Cisd2 reverses most of these dysregulated proteins, indicating that Cisd2 is involved in the regulation of protein homeostasis. Additionally, the ameliorative effects of Cisd2 on protein synthesis could potentially be due to the modulation of the IRS/AKT pathway. IRS/AKT signaling regulates mRNA translation through mTORC1, which in turn activates eIF4E to promote translation. Inhibition of the IRS/AKT signaling in the liver results in hepatic insulin resistance and NAFLD [37]. Two lines of evidence support Cisd2-dependent regulation of Akt (Figure 6B). First, Cisd2 can reverse the increase in the JNK family and PP2A proteins that occurs with age, which facilitates the inactivation of Akt signaling during aging. Second, Cisd2 can suppress the reduction in Ormdl proteins during aging, which results in an inhibition of the Akt pathway by regulation of the ceramide biosynthetic process [30]. Notably, it would seem that Akt might not be the sole contributor to protein homeostasis since Cisd2 haploinsufficiency has been associated with the unfolded protein response [16], another important pathway for protein folding and synthesis.

### 4.6. Activation of the Proteasome-Dependent Degradation Pathway during Aging

Our results show that Hsp70 chaperones are significantly decreased during aging (Figure 6C). Hsp70, with the help of Hsp40 and Hsp110, forms a hydrolytic cycle to prevent misfolding of translating polypeptides. Increased oxidative damage to proteins as well as decreased folding capacity lead to proteasome-mediated degradation of aberrantly misfolded proteins [38,39]. This effect can explain the age-related increase in multiple proteasome subunits, which is remediated by Cisd2TG. The 26S proteasome is composed of the 19S cap and the 20S core. Interestingly, it is important to note that the compensatory induction of proteasome 19S subunits was abrogated in the 26-mo Cisd2TG liver, whereas the increased levels of 20S proteasome subunits were still sustained. Previous studies showed a preference towards the proteolytic machinery of the 20S proteasome under mild oxidative stress [40]. Further studies have demonstrated that the 20S proteasome is capable of degrading oxidized proteins in the absence of ATP and ubiquitin through the interaction between the 20S proteasome and the hydrophobic patches on the oxidized proteins [38]. Whether the selective regulation of proteasome subunits by Cisd2 correlates with the antioxidant function of Cisd2 requires further study.

### 4.7. CISD2 Is a Promising Target for Developing Novel Therapies to Treat NAFLD

Currently, there is no approved drug that capable of specifically treating NAFLD or NASH. The available therapies or interventions for NAFLD treatment include exercise, calorie restriction, gastric bypass surgery and administration of antioxidants [41]. Intriguingly, one of our studies revealed that long-term exercising for eight weeks can enhance Cisd2 expression, as illustrated by the use of Cisd2 luciferase reporter mice [42]. Furthermore, another study by another group also showed that short-term exercising for four weeks can significantly increase the levels of Cisd2 in skeletal muscles and adipose tissue of mice [43]. These findings highlight the potential of Cisd2 as a promising target when developing therapeutic strategies for the treatment of NAFLD and NASH.

## 5. Conclusions

Dysregulated lipid metabolism and fatty liver diseases are among the main risk factors for HCC. Development of therapeutic agents that can bring about effective enhancement of expression of Cisd2 might have potential as a therapeutic strategy for the treatment of fatty liver diseases, thereby preventing subsequent malignant progression to cirrhosis and HCC.

## Data Availability

Not applicable.
